# Color Doppler Imaging Analysis of Retrobulbar Blood Flow Velocities in Primary Open-Angle Glaucomatous Eyes: A Meta-Analysis

**DOI:** 10.1371/journal.pone.0062723

**Published:** 2013-05-13

**Authors:** Nana Meng, Ping Zhang, Huadong Huang, Jinlan Ma, Yue Zhang, Hao Li, Yi Qu

**Affiliations:** 1 Department of Health Care, Qilu Hospital of Shandong University, Jinan, China; 2 Department of Ophthalmology, Qihe Country Hospital, Qihe, China; Saitama Medical University, Japan

## Abstract

**Background:**

To analyze the diagnostic value of color Doppler imaging (CDI) of blood flow in the retrobulbar vessels of eyes with primary open-angle glaucoma (POAG).

**Methods:**

Pertinent publications were retrieved from the Cochrane Central Register of Controlled Trials, PubMed and the ISI Web of Knowledge up to October 2012. Changes in peak systolic velocity (PSV), end diastolic velocity (EDV) and resistive index (RI) of the ophthalmic artery (OA), central retinal artery (CRA) and short posterior ciliary artery (SPCA) of POAG eyes and normal controls were evaluated by CDI. Subgroup analyses were conducted according to whether patients received IOP-lowering drugs treatment and were defined as treated and untreated.

**Results:**

PSV and EDV were statistically significantly reduced in the OA of POAG eyes (*P = *0.0002; *P<*0.00001; respectively), with significant heterogeneity (*P_heterogeneity_<*0.00001, *I^2^* = 94%; *P_heterogeneity_<*0.00001, *I^2^* = 85%; respectively). Similar results were demonstrated for the CRA (*P<*0.00001; respectively) and SPCA (*P* = 0.005; *P<*0.00001; respectively), with significant heterogeneities for both the CRA (*P_heterogeneity_<*0.00001, *I^2^* = 81%; *P_heterogeneity_<*0.00001, *I^2^* = 98%; respectively) and the SPCA (*P_heterogeneity_<*0.00001, *I^2^* = 96%; *P_heterogeneity_<*0.00001, *I^2^* = 93%; respectively). Significant increases in RI were found in all retrobulbar vessels (*P<*0.00001; respectively), with significant heterogeneities (*P_heterogeneity_<*0.00001, *I^2^* = 95%; *P_heterogeneity_<*0.00001, *I^2^* = 94%; *P_heterogeneity_<*0.00001, *I^2^* = 97%; respectively).

**Conclusions:**

This meta-analysis suggests that CDI is a potential diagnostic tool for POAG.

## Introduction

Primary open-angle glaucoma (POAG) is multifactorial in origin and characterized by optic nerve head excavation, visual field defects and psychophysical changes [1]. One hypothesis for the pathogenesis of POAG is that a mechanical mechanism that elevated IOP blocks optic neuronal axoplasmic flow in the madreporite, ultimately leading to apoptosis of the ganglion cell [2]. However, despite therapeutic IOP reduction, some patients still show signs of disease progression, which indicates that other factors might be involved in the glaucomatous damage. Recently, a hypothesis concerning the vasogenic mechanism of POAG was suggested that considers vascular dysregulation [3]–[5]. Inability of the perfusion system to adapt to tissue blood flow requirements or changes in perfusion pressure may lead to chronically low or unstable ocular perfusion [6], which in turn may cause ischemia, oxidative stress or both, possibly leading to glaucomatous damage to the optic nerve head. It seems that vascular factors are important in the development and progression of POAG.

Various techniques have been used to evaluate ocular blood flow in patients with POAG, such as scanning ophthalmoscopy [7], scanning laser Doppler flowmetry [8], [9] and pulsatile ocular blood flow [10], [11]. Compared with these techniques, color Doppler imaging (CDI) has particular advantages in that it is noninvasive, is not affected by poor ocular media, requires no contrast or radiation, and has been used in ophthalmology for 20 years [12], [13]. This ultrasound technique combines simultaneous B-mode ultrasound imaging with colors representing movement based on Doppler frequency shifts. It allows the assessment of blood flow velocities including peak systolic velocity (PSV) and end diastolic velocity (EDV) in the ophthalmic artery (OA), central retinal artery (CRA) and short posterior ciliary artery (SPCA). In addition, resistive index (RI), a measure of peripheral vascular resistance, can be calculated for each retrobulbar vessel.

Numerous studies have reported reduced PSV and EDV and increased RI in the OA, CRA and SPCA in eyes of POAG [14]–[16]. Some studies have found reduced blood flow velocities and increased RI in only one [17]–[19] or two [20], [21] retrobulbar vessels. To the authors’ knowledge, no systematic review of the evidence for the diagnostic potential of CDI in POAG has been published. To obtain a better perspective of the above issues, we performed a meta-analysis of the literature to quantify the value of CDI in measuring the PSV, EDV and RI of the OA, CRA and SPCA in eyes with POAG compared with normal control subjects and to assess the diagnostic value of CDI in POAG.

## Materials and Methods

### Search and Selection

Two researchers independently searched the literature in three databases – the Cochrane Central Register of Controlled Trials, PubMed and the ISI Web of Knowledge – up to October 2012. The search terms were ‘color Doppler imaging or colour Doppler imaging or Doppler ultrasound or CDI’, ‘ocular blood flow or retrobulbar blood flow’ and ‘primary open-angle glaucoma or POAG’ in various combinations, with the language limited to English. The reference lists of case reports, studies and review articles were also reviewed for any additional citations. Publications that involved the use of CDI measurements made in a supine or sitting position in the diagnosis of POAG were included.

### Inclusion Criteria

The definitions of POAG used in the included studies are shown in Table S2. Normal control subjects had IOP≤21 mm Hg, normal optic disc appearance and no visual field defect.

All relevant articles were extracted in print and the final selection was based on discussion among the researchers. Studies were included if they: (i) were randomized clinical controlled trials or observational studies; and (ii) compared blood flow velocities including PSV, EDV and RI in the OA, CRA and SPCA in POAG and normal eyes. When multiple publications from the same study population were available, we checked for duplicate analysis and only the most recent publication was included. For more detailed information, see the PRISMA checklist (Table S1).

### Data Extraction

The following information was extracted by the investigators independently from the published reports, using a standardized protocol and reporting form: first author’s last name, year of publication, country of origin, number of enrolled eyes, mean age of subjects, definition of POAG, type of treatment, and mean change with standard deviations (SD) of measurement indicators.

### Outcome Measures

All studies had PSV and EDV measured by CDI in the OA, CRA and SPCA as major outcomes. PSV (cm/s) was defined as the highest blood flow velocity achieved during systole and was calculated from the frequency of the peak in the Doppler-shifted waveform. EDV (cm/s) was defined as the lowest velocity occurring during diastole and was calculated from the frequency of the trough in the Doppler-shifted waveform. RI was calculated according to the method of Pourcelot [22] as RI = (PSV - EDV)/PSV.

### Statistical Analysis

RevMan software (Review Manager, Version 5.1; The Cochrane Collaboration, 2011) was used for this meta-analysis. For every included study, we calculated the mean difference (MD) for the continuous outcomes (PSV, EDV or RI) along with 95% confidence intervals (95%CIs). The between-study heterogeneity (i.e. the variation in findings not compatible with chance alone) was tested using chi-square based Cochran’s statistics and the inconsistency index (*I^2^*) [23], which indicates the proportion of the variability across studies that is due to heterogeneity rather than sample error. Statistically significant heterogeneity was considered to be present when *P_heterogeneity_<*0.05 and *I^2^* >50%. In the presence of substantial heterogeneity (*I^2^* >50%), a random effect model was adopted as the pooling method; otherwise, a fixed effect model was used. Beggar’s and Egger’s tests were used to determined publication bias [24], [25].

Topical antiglaucoma medications may influence ocular blood flow by increasing flow velocities and reducing RI [26], [27]. Subgroup analyses were conducted according to whether POAG eyes received IOP-lowering drugs and were defined as treated and untreated subgroups. The treated subgroup included eyes that received IOP-lowering drugs and had well controlled IOP (data on duration of medication use were not available); the untreated subgroup included eyes with IOP>21 mm Hg that was newly diagnosed and untreated. All data were displayed as forest plots.

## Results

### Study Characteristics

**Figure 1:**
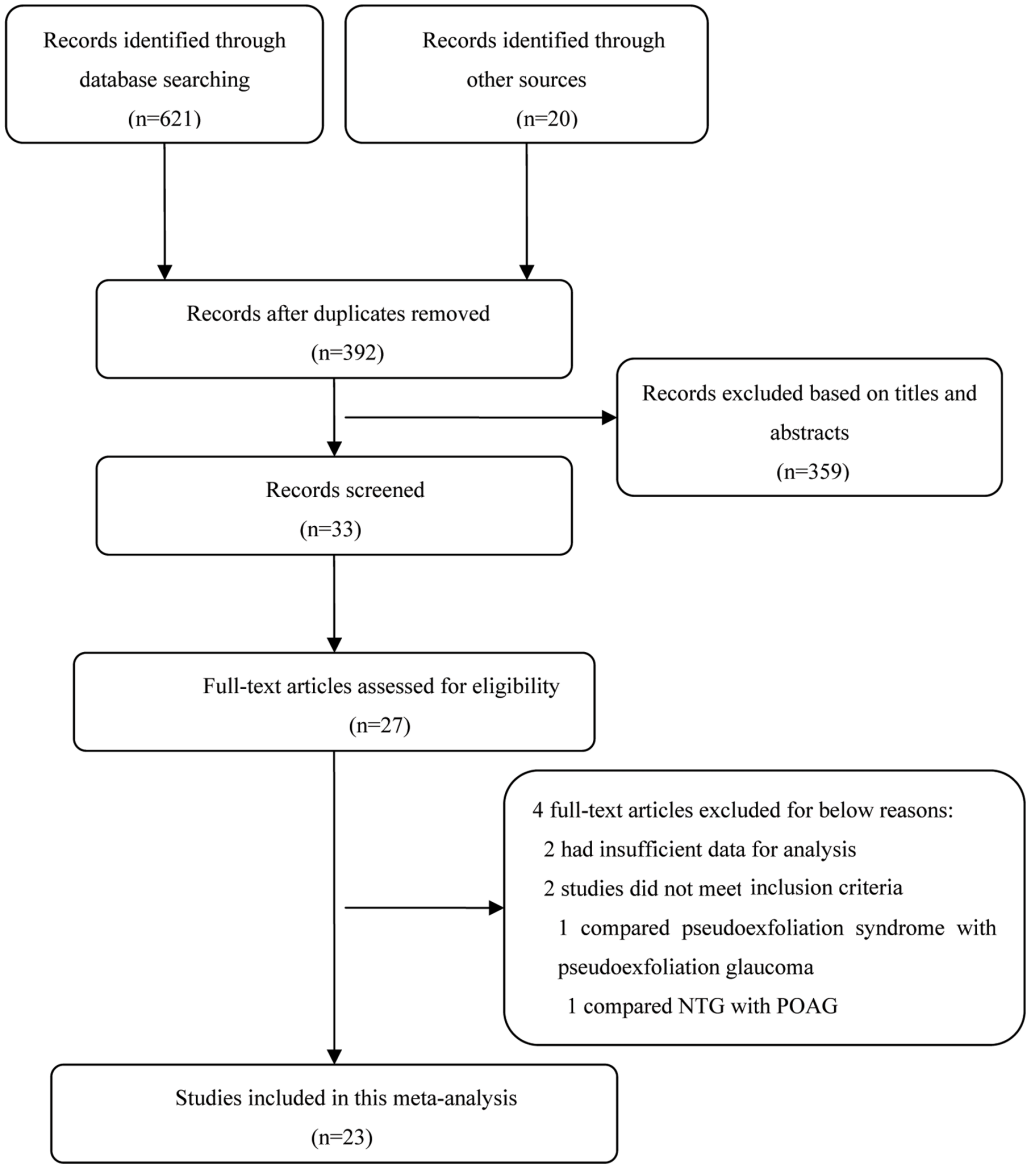
Flowchart of the literature selection process.

Figure 1 summarizes the selection of eligible studies. Twenty-three prospective observational studies of 1286 eyes with POAG and 1052 controls were included in the meta-analysis. Eighteen of the studies concerned POAG eyes that received IOP-lowering drugs and had well controlled IOP [14], [21], [28]–[43]; five studies concerned newly diagnosed and untreated POAG [19], [44]–[47]. CDI was conducted in a sitting position in three studies [31], [40], [44] and in a supine position in the remaining 20 studies [14], [19], [21], [28]–[30], [32]–[39], [41]–[43], [45]–[47]. All of the included studies were of reasonably good quality.

The detailed characteristics of the participants in the 23 studies are given in Table S2. The subjects’ ages ranged from 38.6 to 83.9 years old; the gender distributions of POAG patients and normal control subjects were similar. Clinical heterogeneity was observed in several areas, including the specific characteristics of glaucoma and different treatment protocols. However, these factors were not reported consistently in the publications and hence were not analyzed here.

### Summary Results for PSV, EDV and RI

#### PSV

**Figure 2:**
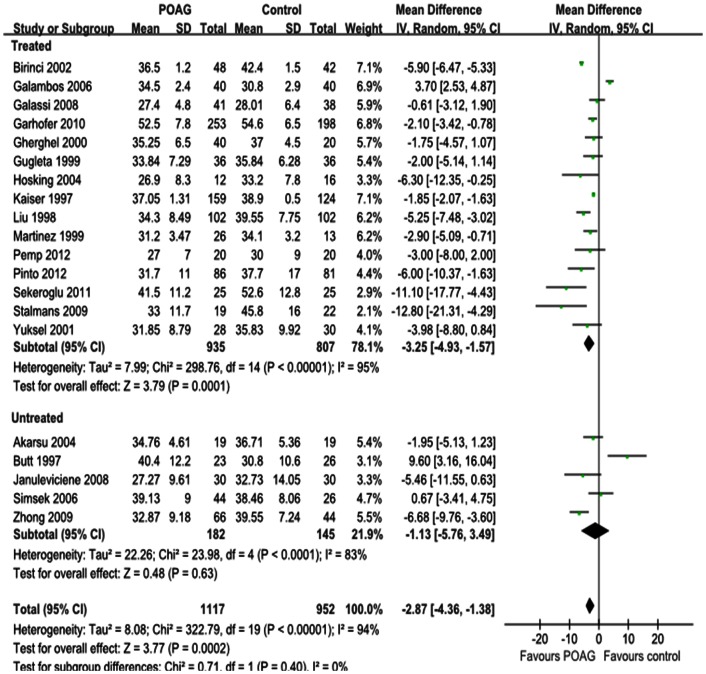
Results of PSV in OA. The forest plot showed the studies’ MDs in PSV in cm/s along with their associated 95%CIs, comparing POAG eyes with normal controls. PSV was significantly decreased in OA in POAG eyes. Subgroup analysis showed that the heterogeneity in OA was due to both the untreated and IOP-lowering drugs treatment subgroup. Negative values favored POAG eyes over normal controls; positive values favored normal controls over POAG eyes.

**Figure 3:**
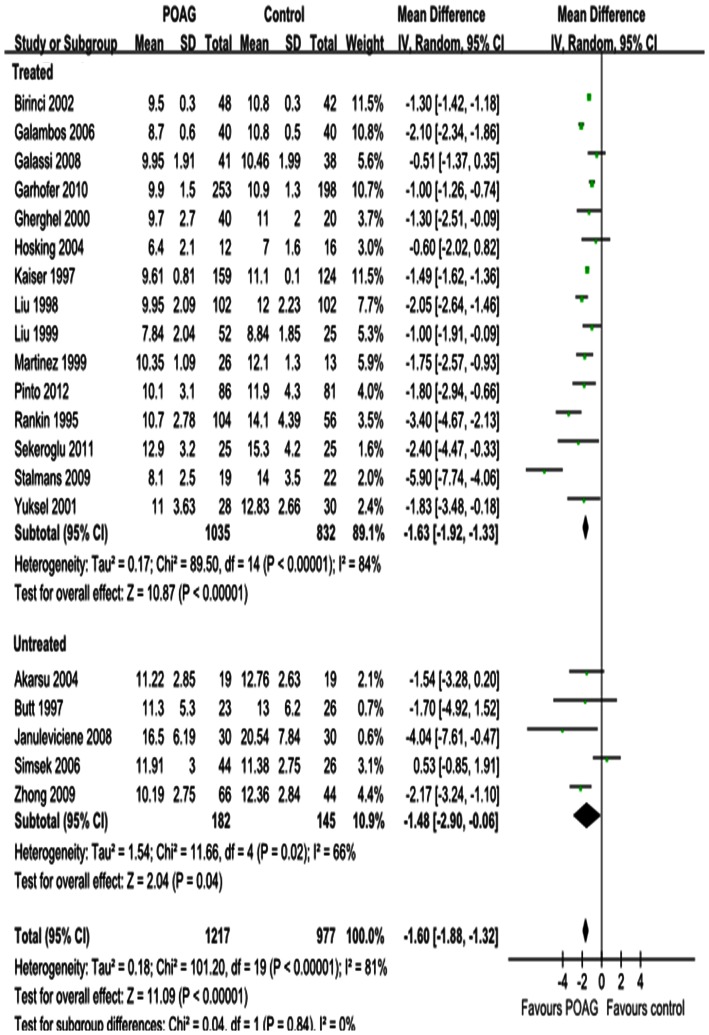
Results of PSV in CRA. PSV was significantly decreased in CRA. Subgroup analysis showed that the heterogeneity in CRA was due to both the untreated and IOP-lowering drugs treatment subgroup.

**Figure 4:**
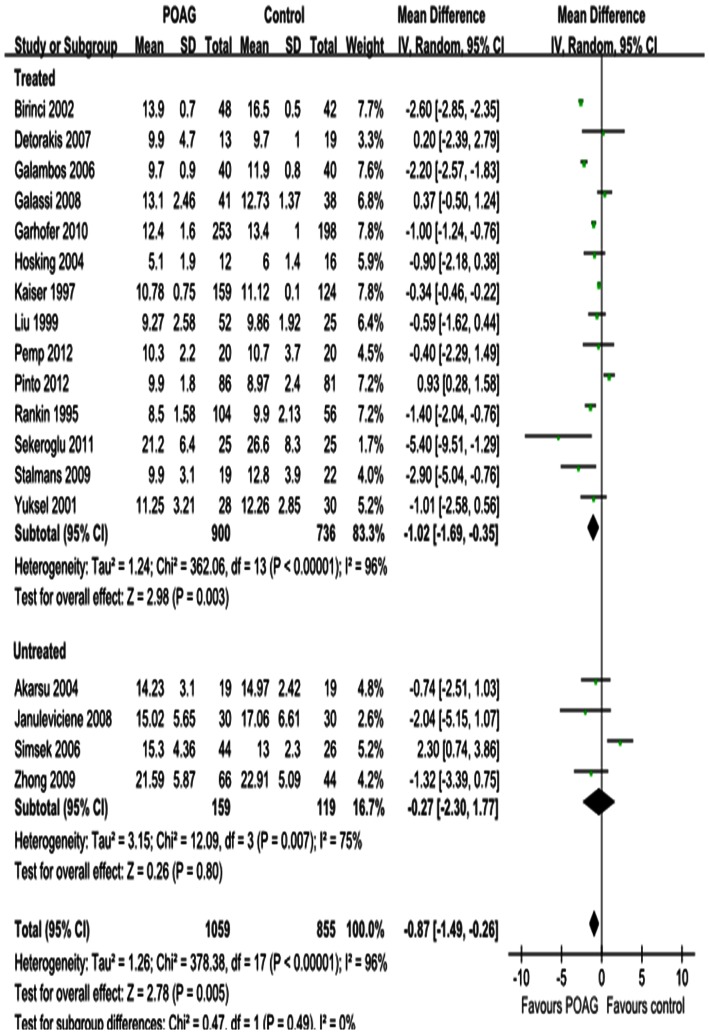
Results of PSV in SPCA. PSV was significantly decreased in SPCA. Subgroup analysis showed that the heterogeneity in SPCA was due to both the untreated and IOP-lowering drugs treatment subgroup.

Differences in the mean change in PSV for each retrobulbar vessel, along with SDs and 95%CIs, are outlined in the forest plot displayed in Figure 2, 3 and 4. The dots represent MDs; the whiskers extending from the dots show the associated 95%CIs. Values on the left side of the vertical line at 0 represent greater changes in PSV in normal controls; values on the right side of the vertical line represent greater changes in eyes with POAG. 95%CIs that do not intersect with the vertical line at 0 indicate the results that were statistically significant at the 0.05 level.

Significant reductions in PSV in the OA and CRA of POAG eyes were found in 20 studies, with a total summary MDs of –2.87 cm/s (95%CI: –4.36 to –1.38, *P = *0.0002; Figure 2) and –1.60 cm/s (95%CI: –1.88 to –1.32, *P<*0.00001; Figure 3) and significant heterogeneities (*P_heterogeneity_<*0.00001, *I^2^* = 94%; *P_heterogeneity_<*0.00001, *I^2^* = 81%; respectively). Significant reductions in PSV in the SPCA of POAG eyes were demonstrated in 18 studies, with a MD of –0.87 cm/s (95%CI: –1.49 to –0.26, *P = *0.005; Figure 4) and significant heterogeneity (P_heterogeneity_ *<*0.00001, I^2^ = 96%). Subgroup analysis of PSV in the OA, CRA and SPCA found significant heterogeneities in both the treated and untreated subgroups (*P_heterogeneity_<*0.00001, *I^2^* = 95% and *P_heterogeneity_<*0.0001, *I^2^* = 83%, Figure 2; *P_heterogeneity_<*0.00001, *I^2^* = 84% and *P_heterogeneity_* = 0.02, *I^2^* = 66%, Figure 3; *P_heterogeneity_<*0.00001, *I^2^* = 96% and *P_heterogeneity_* = 0.007, *I^2^* = 75%, Figure 4).

#### EDV

**Figure 5:**
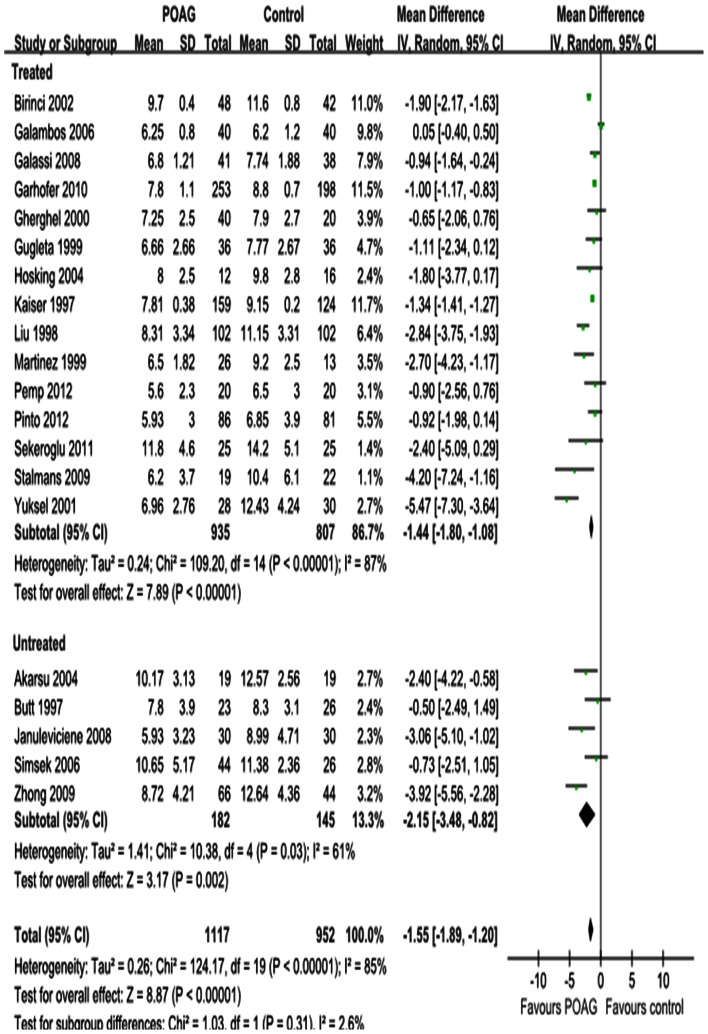
Results of EDV in OA. The forest plot showed the studies’ MDs in EDV in cm/s along with their associated 95%CIs, comparing POAG eyes with normal controls. EDV was significantly decreased in OA in POAG eyes. Subgroup analysis showed the heterogeneity in OA was due to both the untreated and IOP-lowering drugs treatment subgroup. Negative values favored POAG eyes over normal controls; positive values favored normal controls over POAG eyes.

**Figure 6:**
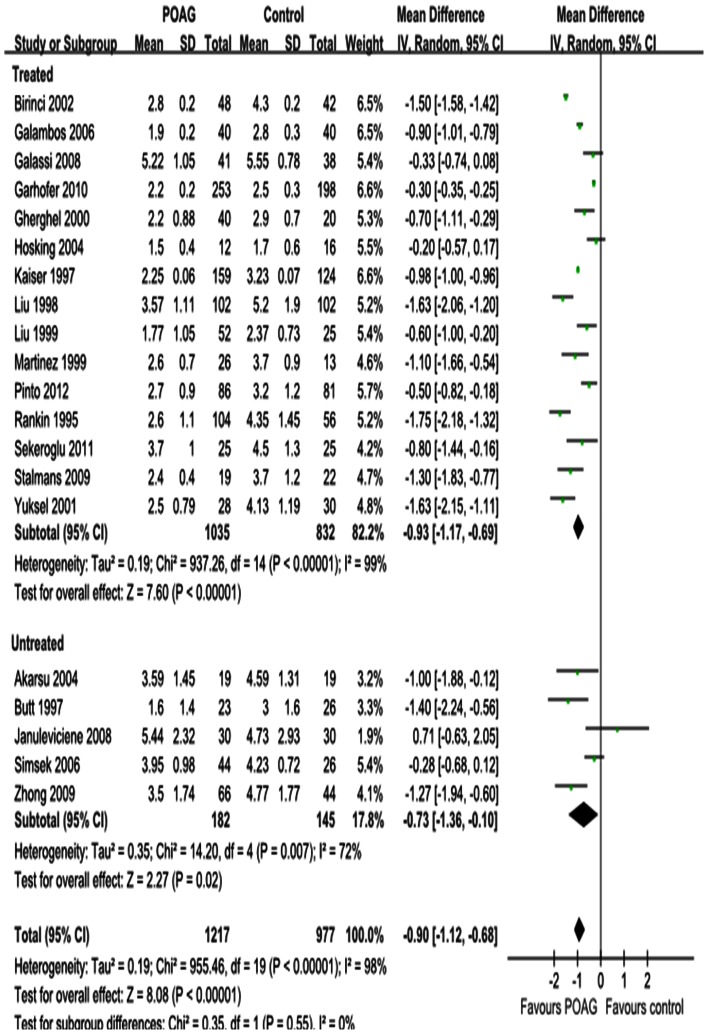
Results of EDV in CRA. EDV was significantly decreased in CRA in POAG eyes. Subgroup analysis showed the heterogeneity in CRA was due to both the untreated and IOP-lowering drugs treatment subgroup.

**Figure 7:**
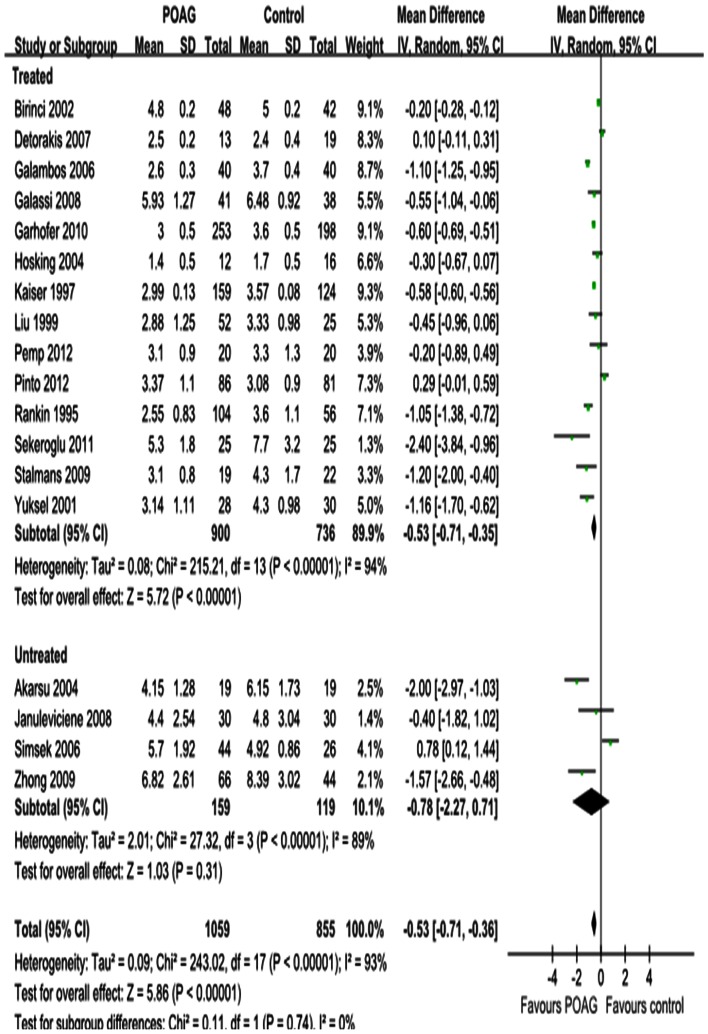
Results of EDV in SPCA. EDV was significantly decreased in SPCA in POAG eyes. Subgroup analysis showed the heterogeneity in SPCA was due to both the untreated and IOP-lowering drugs treatment subgroup.

Figure 5, 6 and 7 show the forest plot for EDV in the OA, CRA and SPCA comparing POAG eyes with controls. Similar to the PSV outcomes, POAG eyes had significantly lower EDV in the OA with a MD of –1.55 cm/s (95%CI: –1.89 to –1.20, *P<*0.00001; Figure 5), in the CRA with a MD of –0.90 cm/s (95%CI: –1.12 to –0.68, *P<*0.00001; Figure 6) and in the SPCA with a MD of –0.53 cm/s (95%CI: –0.71 to –0.36, *P<*0.00001; Figure 7). The heterogeneity was significant for the EDV in each retrobulbar vessel (*P_heterogeneity_<*0.00001, *I^2^* = 85%; *P_heterogeneity_<*0.00001, *I^2^* = 98%; *P_heterogeneity_<*0.00001, *I^2^* = 93%; respectively). Subgroup analysis of EDV in the OA, CRA and SPCA found significant heterogeneities in both the treated and untreated subgroups (*P_heterogeneity_<*0.00001, *I^2^* = 87% and *P_heterogeneit_*_y_ = 0.03, *I^2^* = 6 1%, Figure 5; *P_heterogeneity_<*0.00001, *I^2^* = 99% and *P_heterogeneity_* = 0.007, *I^2^* = 72%, Figure 6; *P_heterogeneity_<*0.00001, *I^2^* = 94% and *P_heterogeneity_<*0.00001, *I^2^* = 89%, Figure 7).

#### RI

**Figure 8:**
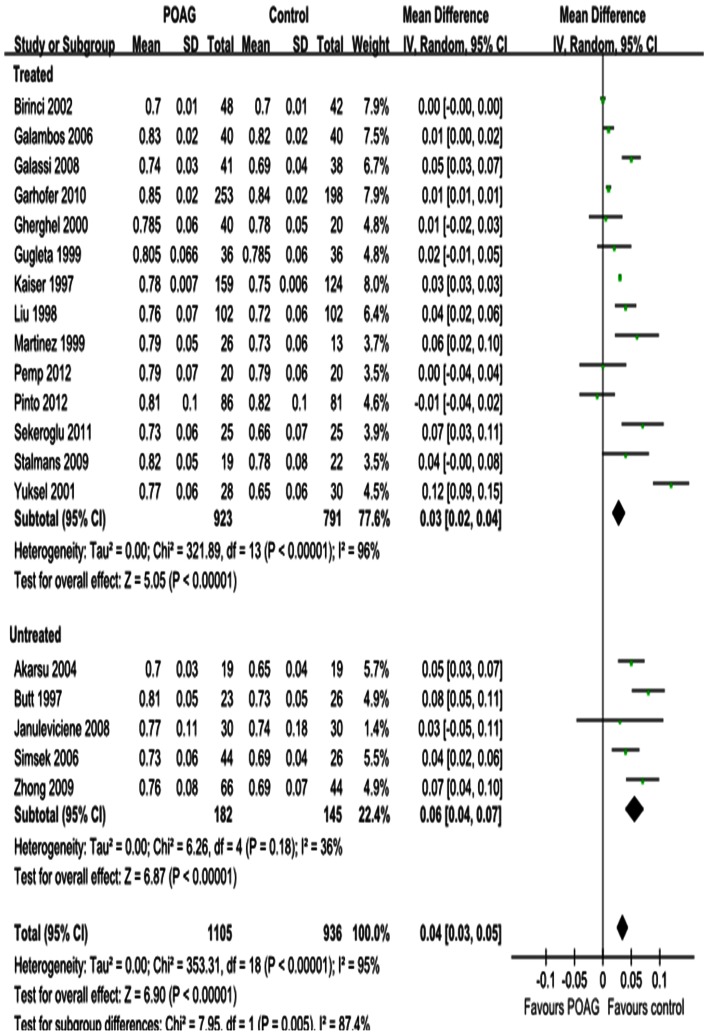
Results of RI in OA. The forest plot showed the studies’ MDs in RI along with their associated 95%CIs, comparing POAG eyes with normal controls. RI was significantly increased in OA in POAG eyes. Subgroup analysis demonstrated the heterogeneity in OA was due to the IOP-lowering drugs treatment. Negative values favored POAG eyes over normal controls; positive values favored normal controls over POAG eyes.

**Figure 9:**
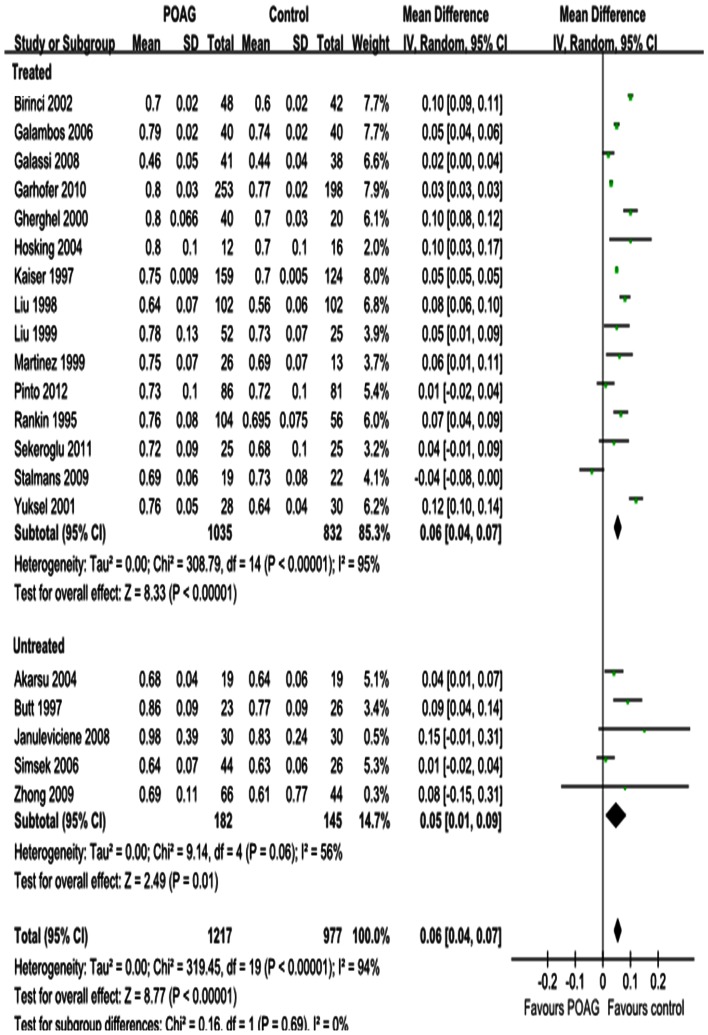
Results of RI in CRA. RI was significantly increased in CRA in POAG eyes. Subgroup analysis demonstrated the heterogeneity in CRA was due to the IOP-lowering drugs treatment.

**Figure 10:**
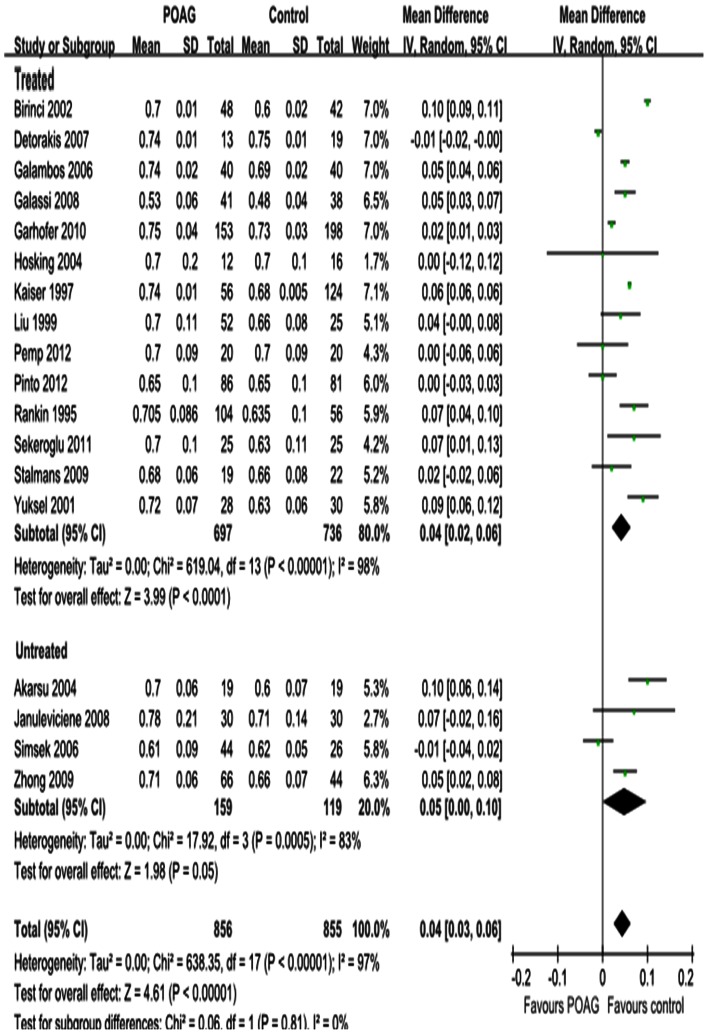
Results of RI in SPCA. RI was significantly increased in SPCA in POAG eyes.

The results for RI in the OA, CRA and SPCA are shown in Figure 8, 9 and 10. Significant increases were observed; the MD was 0.04 in the OA (95%CI: 0.03 to 0.05, *P<*0.00001; Figure 8), 0.06 in the CRA (95%CI: 0.04 to 0.07, *P<*0.00001; Figure 9) and 0.04 in the SPCA (95%CI: 0.03 to 0.06, *P<*0.00001; Figure 10), with significant heterogeneities (*P_heterogeneity_<*0.00001, *I^2^* = 95%; *P_heterogeneity_<*0.00001, *I^2^* = 94%; *P_heterogeneity_<*0.00001, *I^2^* = 97%; respectively). Subgroup analysis demonstrated that the heterogeneities in OA and CRA were due to IOP-lowering drug treatment (*P_heterogeneity_<*0.00001, *I^2^* = 96%; *P_heterogeneity_<*0.00001, I^2^ = 95%; Figure 8 and 9). The heterogeneity in the SPCA was significant in both the treated and untreated subgroups (*P_heterogeneity_<*0.00001, *I^2^* = 98%; *P_heterogeneity = _*0.0005, *I^2^* = 83%; Figure 10).

## Discussion

POAG has long been recognized as the major cause for legal blindness worldwide [48]. For decades, extensive studies have been conducted in attempts to identify the precise pathogenesis of POAG and potential diagnostic methods for its detection.

This meta-analysis was conducted to evaluate the differences in blood flow parameters in the retrobulbar vessels using CDI. CDI measures blood flow velocity but not actual blood flow, because it is impossible to determine accurately the diameter of orbital vessels in vivo with this technique. Despite this limitation, blood flow velocity is probably a good indicator of blood flow within a given vessel [49],[50]. We found that, in eyes with POAG, PSV and EDV in the OA, CRA and SPCA were significantly decreased, whereas the RI in these retrobulbar vessels was significantly increased. The general consensus is that the main blood supply of the anterior optic nerve head is derived from the SPCA, with small contributions from the pial vessels and CRA; the retina derives its blood supply mainly from the CRA. Both the CRA and the SPCA are branches of the OA, which is the main arterial supply to the eyes; therefore, the hemodynamic parameters of the OA, CRA and SPCA reflect local blood supply conditions in the optic disc and retina. PSV reflects the strength of vessel perfusion, whereas EDV reflects the blood perfusion of distal organs and is a sensitive indicator of increased downstream impedance. RI is considered to reflect vascular resistance peripheral to the location where the measurements is made, but is not equivalent to vascular resistance because it depends on both vascular resistance and vascular compliance; only at high vascular compliance is RI an adequate measure of vascular resistance. However, the association of lower EDV with higher RI could be explained by increased vascular resistance [51],[52] and changes in resistance affect diastolic blood flow velocity more than systolic velocity [50], which further aggravates the ischemia of organs. Therefore, decreased blood velocity and increased RI can result in ischemia of the optic disc and, ultimately, glaucomatous damages to the optic nerve head [6].

In this meta-analysis, we found that PSV and EDV were decreased in each retrobulbar vessel in both untreated and IOP-controlled POAG eyes. Considering that the retinal nerve fiber layer in eyes with POAG has been reported to be thinner than that in normal eyes [53],[54], we believe that retrobulbar blood flow reductions affect not only the optic disc, but also the retina. Apoptosis of ganglion cells is probably due to blood flow reduction in the CRA, which is the main artery supplying the inner retina.

The primary limitation of this meta-analysis was that observational studies rather than randomized clinical controlled trials were included. Furthermore, only studies published in English were involved.

Publication bias could have distorted our findings, though no significant publication bias was found. Funnel plots were symmetric (Data not shown) and consistent results were obtained from the Beggar’s and Egger’s tests. Nevertheless, publication bias remains a possibility because studies that report statistically significant results are more likely to be published than those that report non-significant results. Moreover, we did not include unpublished data from conference abstracts, dissertations or pharmaceutical companies.

There was considerable heterogeneity between the studies assessed here, which may be attributable to patient characteristics, types of antiglaucoma medications used, sample size, diagnostic bias, operator experience or CDI devices used. As shown in Table S2, antiglaucoma medications and sample size varied and could have been a source of heterogeneity. The diagnostic criteria for POAG differed among the included studies, which might have led to diagnostic bias; definitions were based on a combination of glaucomatous visual field loss and optic disc abnormalities in most studies, with various criteria and cutoff points (Table S2). In addition, the interpretation of CDI measurements may be operator dependent and it is possible that this is reflected in the heterogeneity. Means of sensitivities, specificities and positive predictive values for different CDI devices are variable and may also resulted in heterogeneity in this meta-analysis. We were unable to conduct regression analysis of the results to explore study-level factors that might have explained some of the heterogeneity.

In summary, our data suggest that blood flow velocities were reduced and RI was increased in all retrobulbar vessels in POAG eyes. Changes of ocular blood flow are important in the development of POAG and CDI is therefore a potential diagnostic tool for this condition.

## Supporting Information

Table S1 **PRISMA checklist.**
(DOC)

Table S2 **characteristics of included studies.**
(DOC)
