# Supplementary material for: Color Doppler Imaging Analysis of Retrobulbar Blood Flow Velocities in Primary Open-Angle Glaucomatous Eyes: A Meta-Analysis
Source: PLoS One. 2013 May 13;8(5):e62723. doi: 10.1371/journal.pone.0062723 (PMC3652862; doi:10.1371/journal.pone.0062723)
Supplement: Table S2 — characteristics of included studies. (DOC) [file pone.0062723.s002.doc]

| Table S2. Characteristics of included studies | | | | | | | | |
| --- | --- | --- | --- | --- | --- | --- | --- | --- |
|  | | | | | | | | |
| **Study** | **Country** | **No.Of Subjects(n) (POAG/Control)** | **Mean Age(years) (POAG/Control)** | **Definition of POAG** | **Type of treatment** | **Results Of PSV(cm/s) (POAG/Control)** | **Results Of EDV(cm/s) (POAG/Control)** | **Results Of RI (POAG/Control)** |
|
| Pemp et al. | Austria | 20/20 | 68±7/66±4 | pathological optic disc appearance | α-Receptor agonists | PSV-OA:27±7/30±9 | EDV-OA:5.6±2.3/6.5±3.0 | RI-OA:0.79±0.07/0.79±0.06 |
| 2012[36] |  |  |  | GVFL | Carbonic anhydrase inhibitors | PSV-SPCA:10.3±2.2/10.7±3.7 | EDV-SPCA:3.1±0.9/3.3±1.3 | RI-SPCA:0.70±0.09/0.70±0.09 |
|  |  |  |  |  | Prostaglandins analogues |  |  |  |
|  |  |  |  |  | β-blockers |  |  |  |
| Pinto et al. | Belgium | 86/81 | 67.4±12/64.6±14 | IOP≥21mmHg | α-Receptor agonists | PSV-OA:31.7±11/37.7±17 | EDV-OA:5.93±3/6.85±3.9 | RI-OA:0.81±0.1/0.82±0.1 |
| 2012[28] |  |  |  | characteristic optic disc damage | Carbonic anhydrase inhibitors | PSV-CRA:10.1±3.1/11.9±4.3 | EDV-CRA:2.7±0.9/3.2±1.2 | RI-CRA:0.73±0.1/0.72±0.1 |
|  |  |  |  | GVFL | Prostaglandins analogues | PSV-SPCA:9.9±1.8/8.97±2.4 | EDV-SPCA:3.37±1.1/3.08±0.9 | RI-SPCA:0.65±0.1/0.65±0.1 |
|  |  |  |  |  | β-blockers |  |  |  |
| Sekeroglu et al. | Turkey | 25/25 | 64.4±8.5/67.3±9.2 | IOP≥21mmHg | α-Receptor agonists | PSV-OA:41.5±11.2/52.6±12.8 | EDV-OA:11.8±4.6/14.2±5.1 | RI-OA:0.73±0.06/0.66±0.07 |
| 2011[37] |  |  |  | characteristic optic disc damage | Carbonic anhydrase inhibitors | PSV-CRA:12.9±3.2/15.3±4.2 | EDV-CRA:3.7±1.0/4.5±1.3 | RI-CRA:0.72±0.09/0.68±0.10 |
|  |  |  |  | GVFL | Prostaglandins analogues | PSV-SPCA:21.2±6.4/26.6±8.3 | EDV-SPCA:5.3±1.8/7.7±3.2 | RI-SPCA:0.70±0.10/0.63±0.11 |
|  |  |  |  |  | β-blockers |  |  |  |
| Garhofer et al. | Austria | 253/198 | 68.1±8.6/68.4±8.5 | pathologic optic disc appearance | α-Receptor agonists | PSV-OA:52.5±7.8/54.6±6.5 | EDV-OA:7.8±1.1/8.8±0.7 | RI-OA:0.85±0.02/0.84±0.02 |
| 2010[14] |  |  |  | GVFL | Carbonic anhydrase inhibitors | PSV-CRA:9.9±1.5/10.9±1.3 | EDV-CRA:2.2±0.2/2.5±0.3 | RI-CRA:0.80±0.03/0.77±0.02 |
|  |  |  |  | ametropia﹤3 diopters | Prostaglandins analogues | PSV-SPCA:12.4±1.6/13.4±1.0 | EDV-SPCA:3.0±0.5/3.6±0.5 | RI-SPCA:0.75±0.04/0.73±0.03 |
|  |  |  |  | anisometropia﹤1 diopter | β-blockers |  |  |  |
| Stalmans et al. | Belgium | 19/22 | 72.3±11.6/68.5±8.9 | IOP≥21mmHg | NA | PSV-OA:33.0±11.7/45.8±16.0 | EDA-OA:6.2±3.7/10.4±6.1 | RI-OA:0.82±0.05/0.78±0.08 |
| 2009[38] |  |  |  | characteristic optic disc damage |  | PSV-CRA:8.1±2.5/14.0±3.5 | EDA-CRA:2.4±0.4/3.7±1.2 | RI-CRA:0.69±0.06/0.73±0.08 |
|  |  |  |  | GVFL |  | PSV-SPCA:9.9±3.1/12.8±3.9 | EDA-SPCA:3.1±0.8/4.3±1.7 | RI-SPCA:0.68±0.06/0.66±0.08 |
| Galassi et al. | Italy | 41/38 | 70.02±6.0/70.3±4.7 | cup-disc ratio:0.6–0.8 | Prostaglandins analogues | PSV-OA:27.4±4.8/28.01±6.4 | EDA-OA:6.8±1.21/7.74±1.88 | RI-OA:0.74±0.03/0.69±0.04 |
| 2008[40] |  |  |  | glaucomous optic disc damage | β-blockers | PSV-CRA:9.95±1.91/10.46±1.99 | EDA-CRA:5.22±1.05/5.55±0.78 | RI-CRA:0.46±0.05/0.44±0.04 |
|  |  |  |  | GVFL |  | PSV-SPCA:13.1±2.46/12.73±1.37 | EDA-SPCA:5.93±1.27/6.48±0.92 | RI-SPCA:0.53±0.06/0.48±0.04 |
| Detorakis | Greece | 13/19 | 61.2±5.1/63.2±1.6 | IOP≥21mmHg | Carbonic anhydrase inhibitors | PSV-SPCA:9.9±4.7/9.7±1.0 | EDV-SPCA:2.5±0.2/2.4±0.4 | RI-SPCA:0.74±0.01/0.75±0.01 |
| et al.2007[30] |  |  |  | characteristic optic disc damage | Prostaglandins analogues |  |  |  |
|  |  |  |  | GVFL | β-blockers |  |  |  |
| Galambos et al. | Germany | 40/40 | 59.5±2.6/60.2±4.2 | IOP≥21mmHg | NA | PSV-OA：34.45±2.4/30.8±2.9 | EDV-OA:6.25±0.8/6.2±1.2 | RI-OA:0.83±0.02/0.82±0.02 |
| 2006[31] |  |  |  | GVFL |  | PSV-CRA:8.7±0.6/10.8±0.5 | EDV-CRA:1.9±0.2/2.8±0.3 | RI-CRA:0.79±0.02/0.74±0.02 |
|  |  |  |  | CD-ratio≥0.6 |  | PSV-SPCA:9.7±0.9/11.9±0.8 | EDV-SPCA:2.6±0.3/3.7±0.4 | RI-SPCA:0.74±0.02/0.69±0.02 |
| Hosking et al. | USA | 12/16 | 59.5±13/60.1±11.1 | IOP≥22mmHg | β-blockers | PSV-OA：26.9±8.3/33.2±7.8 | EDV-OA:8.0±2.5/9.8±2.8 | RI-CRA:0.8±0.1/0.7±0.1 |
| 2004[42] |  |  |  | glaucaomous optic disc damage |  | PSV-CRA:6.4±2.1/7.0±1.6 | EDV-CRA:1.5±0.4/1.7±0.6 | RI-SPCA:0.7±0.2/0.7±0.1 |
|  |  |  |  | GVFL |  | PSV-SPCA:5.1±1.9/6.0±1.4 | EDV-SPCA:1.4±0.5/1.7±0.5 |  |
| Birinci et al. | Turkey | 48/42 | 56.0±3.7/50.6±4.5 | characteristic optic disc damage | β-blockers | PSV-OA:36.5±1.2/42.4±1.5 | EDV-OA:9.7±0.4/11.6±0.8 | RI-OA:0.7±0.01/0.7±0.01 |
| 2002[29] |  |  |  | GVFL | miotic | PSV-CRA:9.5±0.3/10.8±0.3 | EDV-CRA:2.8±0.2/4.3±0.2 | RI-CRA:0.7±0.02/0.6±0.02 |
|  |  |  |  |  |  | PSV-SPCA:13.9±0.7/16.5±0.5 | EDV-SPCA:4.8±0.2/5.0±0.2 | RI-SPCA:0.7±0.01/0.6±0.02 |
| Yuksel et al. | Turkey | 28/30 | 62.8±8.8/63.9±12.1 | IOP≥21mmHg | β-blockers | PSV-OA:31.85±8.79/35.83±9.92 | EDV-OA:6.96±2.76/12.43±4.24 | RI-OA:0.77±0.06/0.65±0.06 |
| 2001[39] |  |  |  | glaucaomous optic disc damage | Carbonic anhydrase inhibitors | PSV-CRA:11.00±3.63/12.83±2.66 | EDV-CRA:2.50±0.79/4.13±1.19 | RI-CRA:0.76±0.05/0.64±0.04 |
|  |  |  |  | GVFL | miotic | PSV-SPCA:11.25±3.21/12.26±2.85 | EDV-SPCA:3.14±1.11/4.30±0.98 | RI-SPCA:0.72±0.07/0.63±0.06 |
| Gherghel et al. | Switzerland | 40/20 | 65.3±9.1/70.6±10.1 | glaucomous optic disc damage | NA | PSV-OA:35.25±6.5/37.0±4.5 | EDV-OA:7.25±2.5/7.9±2.7 | RI-OA:0.79±0.06/0.78±0.05 |
| 2000[32] |  |  |  | GVFL |  | PSV-CRA:9.7±2.7/11.0±2.0 | EDV-CRA:2.2±0.88/2.9±0.7 | RI-CRA:0.8±0.07/0.7±0.03 |
| Gugleta et al. | Switzerland | 36/36 | 60±7.8/59±7.5 | glaucomous optic disc damage | α-Receptor agonists | PSV-OA:33.84±7.29/35.84±6.28 | EDV-OA:6.66±2.66/7.765±2.67 | RI-OA:0.805±0.066/0.785±0.06 |
| 1999[33] |  |  |  | GVFL | Carbonic anhydrase inhibitors | |  |  |
|  |  |  |  |  | Prostaglandins analogues |  |  |  |
|  |  |  |  |  | β-blockers |  |  |  |
|  |  |  |  |  | miotic |  |  |  |
| Liu et al. | China | 52/25 | 62.7±13.8/65.7±11.6 | glaucomous optic disc damage | β-blockers | PSV-CRA:7.84±2.04/8.84±1.85 | EDV-CRA:1.77±1.05/2.37±0.73 | RI-CRA:0.78±0.13/0.73±0.07 |
| 1999[35] |  |  |  | GVFL | miotic | PSV-SPCA:9.27±2.58/9.86±1.92 | EDV-SPCA:2.88±1.25/3.33±0.98 | RI-SPCA:0.70±0.11/0.66±0.08 |
| Martinez et al. | Spain | 26/13 | 64.6±9.9/66.5±9.1 | glaucomous visual field defects | Carbonic anhydrase inhibitors | PSV-OA:31.2±3.47/34.1±3.2 | EDV-OA:6.5±1.82/9.2±2.5 | RI-OA:0.79±0.05/0.73±0.06 |
| 1999[41] |  |  |  |  | β-blockers | PSV-CRA:10.35±1.09/12.1±1.3 | EDV-CRA:2.6±0.7/3.7±0.9 | RI-CRA:0.75±0.07/0.69±0.07 |
| Liu et al. | China | 102/102 | 50.2±14.6/46.9±14.1 | IOP≥21mmHg | NA | PSV-OA:34.3±8.49/39.55±7.75 | EDV-OA:8.31±3.34/11.15±3.31 | RI-OA:0.76±0.07/0.72±0.06 |
| 1998[43] |  |  |  | glaucomous optic disc damage |  | PSV-CRA:9.95±2.09/12±2.23 | EDV-CRA:3.57±1.11/5.2±1.19 | RI-CRA:0.64±0.07/0.56±0.06 |
|  |  |  |  | GVFL |  |  |  |  |
| Kaiser et al. | Switzerland | 159/124 | 64±15.3/58.4±15.7 | glaucomous damage | NA | PSV-OA:37.05±1.31/38.9±0.5 | EDV-OA:7.81±0.38/9.15±0.2 | RI-OA:0.78±0.007/0.75±0.006 |
| 1997[34] |  |  |  |  |  | PSV-CRA:9.61±0.81/11.1±0.1 | EDV-CRA:2.25±0.16/3.23±0.07 | RI-CRA:0.75±0.009/0.70±0.005 |
|  |  |  |  |  |  | PSV-SPCA:10.78±0.75/11.1±0.1 | EDV-SPCA:2.99±0.13/3.57±0.08 | RI-SPCA:0.72±0.02/0.68±0.005 |
| Rankin et al. | Canada | 104/56 | 67.3±12.6/64.9±12.4 | IOP≥21mmHg | β-blockers | PSV-CRA:10.7±2.78/14.1±4.39 | EDV-CRA:2.6±1.1/4.35±1.45 | RI-CRA:0.76±0.08/0.695±0.075 |
| 1995[21] |  |  |  | glaucomous optic disc damage |  | PSV-SPCA:8.5±1.58/9.9±2.13 | EDV-SPCA:2.55±0.83/3.6±1.1 | RI-SPCA:0.705±0.086/0.635±0.1 |
|  |  |  |  | GVFL |  |  |  |  |
| Zhong et al. | China | 66/44 | 57.3±14.1/57.4±12.2 | IOP≥21mmHg | untreated | PSV-OA:32.87±9.18/39.55±7.24 | EDV-OA:8.72±4.21/12.64±4.36 | RI-OA:0.76±0.08/0.69±0.07 |
| 2009[46] |  |  |  | glaucomous optic nerve appearance |  | PSV-CRA:10.19±2.75/12.36±2.84 | EDV-CRA:3.50±1.74/4.77±1.77 | RI-CRA:0.69±0.11/0.61±0.07 |
|  |  |  |  | GVFL |  | PSV-SPCA:21.59±5.87/22.91±5.09 | EDV-SPCA:6.82±2.61/8.39±3.02 | RI-SPCA：0.71±0.06/0.66±0.07 |
| Januleviciene | Lithuania | 30/30 | 58.1±8.6/55.4±9.9 | IOP≥21mmHg | untreated | PSV-OA:27.27±9.61/32.73±14.05 | EDV-OA:5.93±3.23/8.99±4.71 | RI-OA:0.77±0.11/0.74±0.18 |
| et al. 2008[44] |  |  |  | glaucomous optic nerve appearance |  | PSV-CRA:16.50±6.19/20.54±7.84 | EDV-CRA:5.44±2.32/4.73±2.93 | RI-CRA:0.98±0.39/0.83±0.24 |
|  |  |  |  | GVFL |  | PSV-SPCA:15.02±5.65/17.06±6.61 | EDV-SPCA:4.40±2.54/4.80±3.04 | RI-SPCA：0.78±0.21/0.71±0.14 |
| Simsek et al. | Turkey | 44/26 | 55.8±9.5/51.3±5.6 | IOP≥21mmHg | untreated | PSV-OA:29.13±9 /38.46±8.06 | EDV-OA:10.65±5.17/11.38±2.36 | RI-OA:0.73±0.06/0.69±0.04 |
| 2006[47] |  |  |  | glaucomous optic disc damage |  | PSV-CRA:11.91±3/11.38±2.75 | EDV-CRA:3.95±0.98/4.23±0.72 | RI-CRA:0.64±0.07/0.63±0.06 |
|  |  |  |  | GVFL |  | PSV-SPCA:15.3±4.36/13±2.3 | EDV-SPCA:5.7±1.92/4.92±0.86 | RI-SPCA：0.61±0.09/0.62±0.05 |
| Akarsu et al. | Turkey | 19/19 | 51.5±12.9/52.4±10.9 | IOP≥22mmHg | untreated | PSV-OA:34.76±4.61/36.71±5.36 | EDV-OA:10.17±3.13/12.57±2.56 | RI-OA:0.70±0.03/0.65±0.04 |
| 2004[45] |  |  |  | glaucomous visual field defects |  | PSV-CRA:11.22±2.85/12.76±2.63 | EDV-CRA:3.59±1.45/4.59±1.31 | RI-CRA:0.68±0.04/0.64±0.06 |
|  |  |  |  | optic disc excavation |  | PSV-SPCA:14.23±3.10/14.97±2.42 | EDV-SPCA:4.15±1.28/6.15±1.73 | RI-SPCA：0.70±0.06/0.60±0.07 |
| Butt et al. | Scotland | 23/26 | 69.0±8.0/65.7±6.0 | IOP≥25mmHg | untreated | PSV-OA:40.4±12.2/30.8±10.6 | EDV-OA:7.8±3.9/8.3±3.1 | RI-OA:0.81±0.05/0.73±0.05 |
| 1997[19] |  |  |  | glaucomous optic disc damage |  | PSV-CRA:11.3±5.3/13.0±6.2 | EDV-CRA:1.6±1.4/3.0±1.6 | RI-CRA:0.86±0.09/0.77±0.09 |
|  |  |  |  | GVFL |  |  |  |  |

POAG=Primary Open-angle Glaucoma; IOP= Intraocular Pressure; PSV= Peak Systolic Velocity; EDV= End Diastolic Velocity; RI= Resistive Index; OA=Ophthalmic Artery; CRA=Central Retinal Artery; SPCA= Short Posterior Ciliary Artery; GVFL=Glaucomatous visual field loss; NA=Not available.
